# Diagnostic Value of MAML2 Rearrangements in Mucoepidermoid Carcinoma

**DOI:** 10.3390/ijms23084322

**Published:** 2022-04-13

**Authors:** Julia C. Thierauf, Alex A. Farahani, B. Iciar Indave, Adam Z. Bard, Valerie A. White, Cameron R. Smith, Hetal Marble, Martin D. Hyrcza, John K. C. Chan, Justin Bishop, Qiuying Shi, Kim Ely, Abbas Agaimy, Maria Martinez-Lage, Vania Nose, Miguel Rivera, Valentina Nardi, Dora Dias-Santagata, Salil Garg, Peter Sadow, Long P. Le, William Faquin, Lauren L. Ritterhouse, Ian A. Cree, A. John Iafrate, Jochen K. Lennerz

**Affiliations:** 1 Department of Pathology, Center for Integrated Diagnostics, Massachusetts General Hospital and Harvard Medical School, Boston, MA 02114, USA; jthierauf@mgh.harvard.edu (J.C.T.); afarahani@mgh.harvard.edu (A.A.F.); azbard@mgh.harvard.edu (A.Z.B.); hetalvdesai@gmail.com (H.M.); mnrivera@mgh.harvard.edu (M.R.); vnardi@partners.org (V.N.); ddiassantagata@mgh.harvard.edu (D.D.-S.); sgarg1@partners.org (S.G.); long.le@mgh.harvard.edu (L.P.L.); lritterhouse@mgh.harvard.edu (L.L.R.); aiafrate@partners.org (A.J.I.); 2 Department of Otorhinolaryngology, Head and Neck Surgery, Heidelberg University Hospital and Research Group Molecular Mechanisms of Head and Neck Tumors, German Cancer Research Center (DKFZ), 69120 Heidelberg, Germany; 3 International Agency for Research on Cancer (IARC), World Health Organization, 69372 Lyon, France; indavei@iarc.fr (B.I.I.); whitev@iarc.fr (V.A.W.); creei@iarc.fr (I.A.C.); 4 Department of Pathology, Massachusetts General Hospital and Harvard Medical School, Boston, MA 02114, USA; cameron.ray.smith@gmail.com (C.R.S.); mmartinez-lage@mgh.harvard.edu (M.M.-L.); vnose@mgh.harvard.edu (V.N.); psadow@mgh.harvard.edu (P.S.); wfaquin@mgh.harvard.edu (W.F.); 5 Department of Pathology and Laboratory Medicine, University of Calgary, Calgary, AB 2500, Canada; mdhpath@gmail.com; 6 Department of Pathology, Queen Elizabeth Hospital, Kowloon, Hong Kong, China; jkcchan@netvigator.com; 7 Department of Pathology, University of Texas Southwestern Medical Center, Dallas, TX 75390, USA; justin.bishop@utsouthwestern.edu; 8 Department of Pathology, Emory University Hospital, Atlanta, GA 30322, USA; qshi30@emory.edu; 9 Department of Pathology, Vanderbilt University Medical Center, Nashville, TN 37232, USA; kim.ely@vumc.org; 10 Institute of Pathology, Friedrich Alexander University Erlangen-Nürnberg, University Hospital, 91054 Erlangen, Germany; abbas.agaimy@uk-erlangen.de

**Keywords:** biomarker, mucoepidermoid, adenosquamous, molecular testing, next-generation sequencing, fusion gene, CRTC

## Abstract

Mucoepidermoid carcinoma (MEC) is often seen in salivary glands and can harbor MAML2 translocations (MAML2+). The translocation status has diagnostic utility as an objective confirmation of the MEC diagnosis, for example, when distinction from the more aggressive adenosquamous carcinoma (ASC) is not straightforward. To assess the diagnostic relevance of MAML2, we examined our 5-year experience in prospective testing of 8106 solid tumors using RNA-seq panel testing in combinations with a two-round Delphi-based scenario survey. The prevalence of MAML2+ across all tumors was 0.28% (*n* = 23/8106) and the majority of MAML2+ cases were found in head and neck tumors (78.3%), where the overall prevalence was 5.9% (*n* = 18/307). The sensitivity of MAML2 for MEC was 60% and most cases (80%) were submitted for diagnostic confirmation; in 24% of cases, the MAML2 results changed the working diagnosis. An independent survey of 15 experts showed relative importance indexes of 0.8 and 0.65 for “confirmatory MAML2 testing” in suspected MEC and ASC, respectively. Real-world evidence confirmed that the added value of MAML2 is a composite of an imperfect confirmation test for MEC and a highly specific exclusion tool for the diagnosis of ASC. Real-world evidence can help move a rare molecular-genetic biomarker from an emerging tool to the clinic.

## 1. Introduction

**Figure 1:**
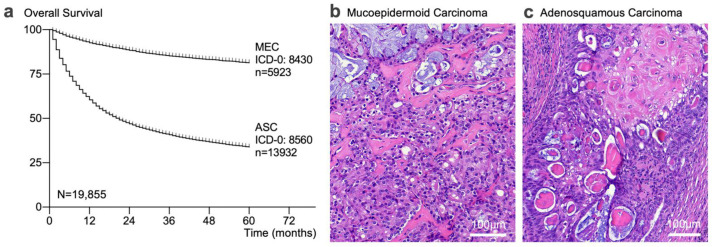
Survival Analysis and Morphology of Mucoepidermoid Carcinoma (MEC) and Adenosquamous Carcinoma (ASC). (**a**) Data were accessed using the SEER database comprised of 18 registries from 2000–2017 from the National Cancer Institute. Only patients with mucoepidermoid carcinoma (MEC, ICD-O-8430) and adenosquamous carcinoma (ASC, ICD-O-8560) were included in this analysis. Reports of observed survival were pulled as case listings and analyzed using the Kaplan–Meier method. (**b**) Mucoepidermoid carcinoma (MEC; MAML2+). (**c**) Adenosquamous carcinoma (ASC; MAML2–) with keratinizing and non-keratinizing squamous and glandular components. Abbreviations: ASC, adenosquamous carcinoma; MEC, mucoepidermoid carcinoma.

The diagnostic distinction between adenosquamous carcinoma (ASC) and mucoepidermoid carcinoma (MEC) is clinically relevant because ASC is a more aggressive disease and patients with ASC have shorter overall survival times (Figure 1a) [1,2,3,4,5,6]. Briefly, MEC is the most common malignant salivary gland neoplasm [7,8], the tumor grows in cystic and solid patterns, and is composed of three cell types in variable combinations: squamous-like, intermediate (including clear cell), and mucinous tumor cells (Figure 1b) [9,10]. However, in any single sample, one cell type may be minor or absent, and many tumors are even composed of a single cell type, usually intermediate cells (so-called monomorphic MEC variants). In contrast, ASC is rare and composed of only two cell types: malignant keratinizing or non-keratinizing squamous cells and glandular cells [11] with at least 10% of each (Figure 1c) [10,12,13]. Morphologically, the absence of a third (intermediate) cell type in a high-grade tumor and the presence of keratinization point toward ASC [12,14]. In most cases, the histopathological distinction is straightforward; however, the morphological overlap is substantial and, historically, ASC has been considered the same entity as MEC [15,16]. A diagnostic distinction in occasional cases, or when tissue is limited (e.g., biopsy or fine-needle aspiration specimen) can be very challenging [11], underscoring the need for diagnostic biomarkers. 

Molecular genetic studies have identified that MECs can harbor rearrangements of the MAML2 (Mastermind-Like Transcriptional Coactivator 2) [17] locus in 35–77% of cases [18,19,20]. Briefly, the protein encoded by the MAML2 gene is a member of the mastermind-like family of proteins. MAML2 is proline- and glutamine-rich and contains a conserved basic domain that binds the ankyrin repeat domain of the intracellular domain of the Notch receptors (ICN1-4) in their N-terminus and a transcriptional activation domain in their C-terminus. Molecularly, MAML2 acts as a transcriptional coactivator for NOTCH proteins [17]. Functionally, MAML2 and NOTCH signaling is context dependent; however, NOTCH signaling is oncogenic in several solid tumors [17]. The MAML2 fusion gene encodes a chimeric protein in which the N-terminal region of the CRTC1 that binds CREB replaces the N-terminal basic domain at MAML2, which results in ligand-independent activation of NOTCH target genes [19]. Diagnostically, many studies emphasize the absence of MAML2 rearrangements in ASC [11,18,19,20,21,22,23], implying that the detection of the rearrangement is a diagnostic biomarker for MEC. Notably, despite the inclusion of the MAML2 status as a distinguishing feature in the WHO tumor classification [15], studies comparing the frequency of MAML2 rearrangements in MEC and ASC are limited [18,19,20,22,23]. These studies use a combination of break-apart FISH and/or RT-PCR to examine MAML2 rearrangements in salivary [23], pulmonary [18,20], thymic [22], and cervical [19] MEC and ASC and show the presence of MAML2 rearrangement in 51% of MEC and absence in ASC. Importantly, we are not aware of any report describing MAML2-rearranged ASC [24], supporting the notion that the presence of MAML2 rearrangements rules out ASC. These data indicate that the absence of MAML2 could become definitional for ASC [10], especially in diagnostically challenging cases.

The value of a diagnostic biomarker is related to the context of use. However, with rare entities, the delineation of the specific, context-dependent diagnostic value emerges through mounting published evidence and utilization. Concerning MAML2, studies that applied a framework to align real-world evidence from clinical utilization with the context-dependent perceived value of MAML2 in distinguishing ASC from MEC are rare [25].

Here, we performed a comprehensive review of MAML2 rearrangements encountered as part of our routine clinical testing experience, including the frequency across all tested tumor types and diagnostic performance in MEC and ASC. We also examined the timing of the MAML2 test order and results from the integration of the molecular data into the surgical pathology context and modeled the added value of molecular testing to support diagnostic decision-making. We derived key clinical scenarios and solicited expert opinion using the Delphi method to quantify the added value of MAML2 testing. Determining the relative importance of a diagnostic biomarker in distinct clinical scenarios is paramount when determining the clinical utility of MAML2 rearrangement as a diagnostic biomarker. The proposed framework may serve as a blueprint for the assessment of other rare diagnostic biomarkers.

## 2. Results

### 2.1. MAML2 Fusions Were Rare Outside of Major Salivary Gland Tumors 

We determined the overall frequency of MAML2 rearrangements in a total of *n* = 8106 analytical cases as 0.28% (*n* = 23/8106, Table 1 and Table S1). The NGS-based RNA fusion sequencing assay was applied as part of an institutional screening program across solid cancers (Table S1). The assay allowed for the identification of the MAML2 fusion partner, and we found the following five fusion gene partners: CRTC1 (*n* = 17, head and neck; *n* = 2 breast), CRTC3 (*n* = 1, pharyngeal mass), KMT2A (*n* = 1, thymus), YAP1 (*n* = 1, brain), and SAMSN1 (*n* = 1, lung); the exons are shown in Table 1 and Table S2. Analysis by primary site showed that the majority of MAML2 rearranged cases were found in head and neck tumors (*n* = 18/23, 78.3%) with an overall frequency of 5.86% (*n* = 18/307, Table 1). By anatomic subsite, most MAML2+ cases were found in major salivary glands (*n* = 17, Tables S1 and S2). Only five MAML2 rearranged tumors were found outside the head and neck region (0.06%, Table 1 and Table S2) and a review of the medical record showed no prior or subsequent diagnosis of a salivary gland neoplasm in these patients. Notably, the final diagnosis was revised to MEC in both MAML2+ breast cases whereas the histology in the three other MAML2+ tumors was incompatible with MEC (Table S2). Thus, MAML2 rearrangements were found primarily in salivary gland tumors with a small subset of MAML2+ tumors with unusual histology and uncommon fusion partners outside the head and neck region.

### 2.2. Test Order Analysis of MAML2 in Diagnostic Practice

**Figure 2:**
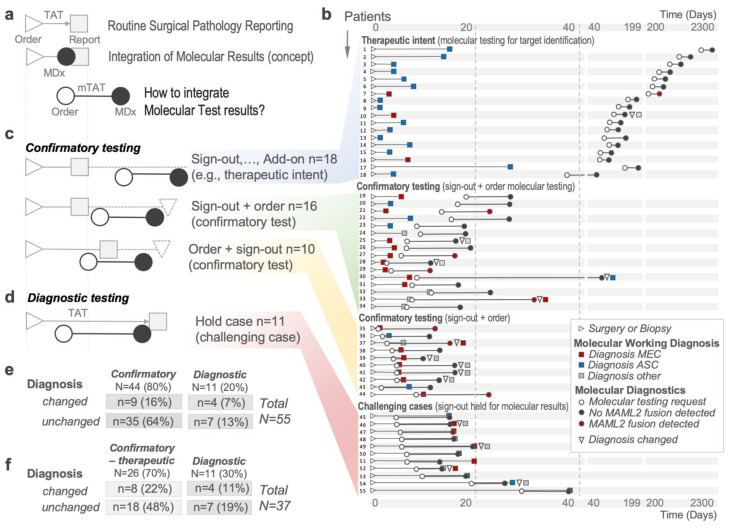
Molecular–genetic diagnostic test results integration. The generation of a diagnosis from a tissue sample relies on the interpretation carried out by the surgical pathologist. To follow the concept of integrating molecular diagnostics, the time of molecular testing (mTAT) needs to be considered. To perform molecularly informed decision-making to support the correct diagnosis, the molecular result must be available before surgical pathology sign out. Integration of molecular test results into the surgical pathology diagnosis results in several scenarios. (**a**) To obtain a diagnosis, the tissue specimen is submitted to the surgical pathologist and will be analyzed in a certain turn-around time (TAT). (**b**) Time points of surgical pathology and molecular order and surgical pathology and molecular sign-out for patients. Red squares represent the working diagnosis MEC, blue squares represent the working diagnosis ASC, and grey squares indicate another working diagnosis. White circles represent the time point of the molecular testing request, red circles show a detected MAML2 rearrangement, and blue circles indicate a negative (no MAML2 rearrangement) result. White horizontal triangles indicate the time point of surgical pathology order and are normalized to 0, and white vertical triangles mark a changed surgical diagnosis after molecular testing. (**c**) In a confirmatory intent, molecular testing is usually ordered right before or shortly after the case is diagnosed (signed out). (**d**) When confirmation of a diagnosis requires molecular diagnostics, the case is held until the molecular result is available to the surgical pathologist. Light grey squares indicate the final diagnosis, white circles mark molecular test ordering, and black circles show molecular testing results. (**e**,**f**) Four-field tables with cases represented according to their order intent (confirmatory vs. diagnostic) and whether their final diagnosis was changed due to molecular testing. Abbreviations: MDx, molecular diagnostics; mTAT, molecular turn-around time; TAT, turn-around time.

The concept of molecularly informed diagnostics is compelling (Figure 2a); however, the real-world test order practice in a consecutive series of 55 cases where the molecular working diagnosis entailed MEC or ASC looks fundamentally different (Figure 2b, Table S3). Vastly oversimplified, the molecular turn-around time requirements impose an important operational decision on the surgical pathologist, namely, can the case be finalized without molecular data (Figure 2c, confirmatory testing) or are molecular results needed for the initial surgical pathology report (Figure 2d, diagnostic testing)? In the diagnostic subset, the breakdown by molecular working diagnosis was roughly even (*n* = 5 ASC vs. *n* = 4 MEC vs. 2 unknown). In contrast, the confirmatory subset showed considerable heterogeneity. We noticed three distinct order patterns (Figure 2b). We identified 18 cases where molecular testing was ordered more than 30 days after the final surgical pathology diagnosis by our oncologists (i.e., to identify therapeutic targets). In this subgroup, we noticed a high rate of ASC (83%, *n* = 15/18). Conversely, cases where molecular testing was ordered and the surgical pathology case was signed out before molecular results were available showed a high fraction of MEC (70%, *n* = 7/10; Figure 2b). We considered these trends quantitative manifestations of how molecular testing is applied in our practice. In our setting, the observed ratio for confirmatory vs. diagnostic intent was ~4:1 (*n* = 44 vs. *n* = 11, Figure 2e), or ~2.4:1 when subtracting the subset of 18 cases where molecular testing was ordered for the identification of therapeutic targets (*n* = 26 vs. *n* = 11, Figure 2f).

We next examined the breakdown by tumor type. In cases with the molecular working diagnosis ASC, we noticed a predominance of confirmatory testing (80%, *n* = 20) vs. diagnostic testing (20%, *n* = 5). Following prior publications [26], MAML2 rearrangements were absent in ASC. In cases with the molecular working diagnosis of MEC, molecular testing was predominantly ordered for confirmation (83%, *n* = 19/23), and in the confirmatory subset, MAML2 rearrangements were detected in 42% (*n* = 8/19). In MEC cases held for surgical pathology sign-out, MAML2 was detected in 25% (*n* = 1/4). Thus, in the subset of cases used for test order analysis, the overall frequency of MAML2 rearrangements in cases with the working diagnosis of MEC was 39% (*n* = 9/23), which was somewhat lower than in prior reports [27,28,29]. 

The lower MAML2 rearrangement frequency in MEC raised questions about the type of cases submitted for molecular testing in our practice. A query in the surgical pathology database revealed a slightly higher rate of molecular testing in ASC (29%; *n* = 25/85) vs. MEC (21%, *n* = 23/108); however, this trend did not reach statistical significance (*p* = 0.18, Fisher’s exact test). A manual review of all 110 potential MEC cases and contingency analysis revealed no significant clinicopathological differences with one notable exception: MEC cases submitted for molecular testing showed a significantly larger fraction of high-grade tumors (32%, *n* = 6/19) when compared to those not submitted for molecular testing (10%, *n* = 7/70; *p* = 0.028; Table S4). This may explain the lower MAML2+ rate. Accordingly, and similar to prior reports, we observed a higher rate of MAML2 gene fusions in tumors with lower grade (83%) when compared to higher grade MEC (16%; *p* = 0.04; Table S4). 

### 2.3. Diagnostic Performance Assessment

The frequency of identifying MAML2 rearrangements has concrete test performance implications [30]; as such, we compared test performance characteristics across several relevant diagnostic settings (Table 2). First, when considering the differential diagnosis of MEC vs. ASC, MAML2 rearrangements were 100% specific for MEC. In our dataset of salivary gland malignancies, we observed 60% sensitivity (Table 2 and Table S5). When comparing the diagnostic performance assessment of MAML2 for the distinction of MEC from ASC in our test order analysis, we noted the lowest diagnostic performance (i.e., sensitivity) in the most diagnostic challenging cases.

### 2.4. Added Value of MAML2 Testing and Economic Impact Analysis

**Figure 3:**
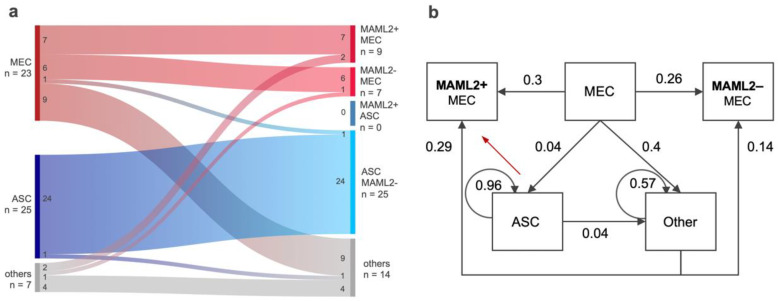
State transition probability. (**a**) Alluvial diagram to represent changes from working diagnosis before molecular testing to final diagnosis. The states MEC, MAML2+, MAML2−, ASC, and others were assigned as variables to parallel vertical axes. The values represent the total number of cases in each group before and after molecular diagnoses. The height of each block represents the size of the cluster, and the height of each stream field represents the size of the components contained in both blocks connected by the stream field. (**b**) Pre- and post-molecular fusion analysis diagnoses represent states in a memoryless (Markov) stochastic process where state transitions (arrows) are determined exclusively by the relationship between the pre-molecular diagnoses and the post-molecular fusion analysis diagnoses. The empirical estimates of state transition probabilities were computed by simply conditioning and normalizing the data. Given the size of our data set, relative to that of the state space, these empirical estimates were not expected to converge to the true values of the underlying state transition probabilities, but rather represent our observations via pure summary statistics. Numbers indicate the probability of transitions from the indicated states to another, with the 3 states of working diagnosis *MEC, ASC, and others* and 4 states at final diagnosis (*MAML2+ MEC, MAML2− MEC, ASC, and other*). The transition from ASC to MAML2+ MEC did not exist and is therefore marked with a red arrow. Abbreviations: ASC, adenosquamous carcinoma; MEC, mucoepidermoid carcinoma.

We plotted the test order analysis data in an alluvial diagram to illustrate state transitions (Figure 3a). The diagram shows that the working diagnoses of ASC, MEC, and others differed in terms of the probability of a state transition. Specifically, when considering that molecular test results contributed to *n* = 14 shifts across the three diagnostic groups (MEC, ASC, other), as well as positively confirmed *n* = 7 MAML2+ MEC cases (no change in diagnosis), the benefit of MAML2 in this setting was at least 38% (*n* = 21 of 55 cases), or up to 81% when also considering the 24 MAML2-confirmed ASC cases. In other words, molecular testing for MAML2 had added value in at least 1 case per 2.6 cases tested.

To estimate the cost savings of our molecular testing practice, we compared the estimated total cost for genotyping all 193 ASC and MEC diagnoses (USD 115,396.63) with the number of performed tests (*n* = 55, USD 32,885.05). These numbers showed that cognizant use of molecular testing by our surgical pathologists amounted to USD 82,511.58 in cost savings (71.5% cost reduction). When considering that 81% of performed tests demonstrated added value (USD 26,095.95), the estimated cost of unnecessary tests was rather low USD 5979.10 (only 5% of the cost), which we considered meaningful when used in clinical practice.

### 2.5. A Markov Model for MAML2-Related Diagnostic State Transition Probabilities 

Mathematically, the molecular working diagnosis and final diagnosis can be viewed as states where the MAML2 test result (positive or negative) influences the state transition probability. For example, when encountering the working diagnosis ASC (*n* = 25 cases, Figure 3a), the empirical estimates for the state transition probability of keeping the diagnosis ASC was 0.96 (*n* = 24 cases) and the probability of diagnosing something other than MEC or ASC was 0.04 (*n* = 1 case). For the final model, we calculated sample estimates for the probability of transitioning from state to state and integrated all data into an estimate for the Markov chain state transition probability matrix (Figure 3b). The matrix depicts the specific pre-test probabilities by working diagnosis that must be normalized (e.g., working diagnosis MEC: 0.4 + 0.3 + 0.26 + 0.04 = 1; Figure 3b). We noted that certain transitions were not encountered (e.g., ASC to MAML2+ MEC; Figure 3b, red arrow) and, therefore, remain without estimates.

### 2.6. Specifying the Relative Diagnostic Value of MAML2 via an Expert Survey

Based on our test order analysis, we selected seven relevant MAML2 testing scenarios (Table 3 and Table S6) and assessed the relative diagnostic value by administering an expert survey (see the Supplementary Materials). Of the 18 contacted pathologists, the survey was completed by 15 board-certified pathologists. Breakdown by subspecialty of the respondents showed: head and neck pathology (47%), molecular pathology (33%), or another subspecialty (20%). 

The group had, as stated via self-reporting, a cumulative experience of 250 years (range: 4–40 years, median: 14 years; Table S7). Following the 10,000 h rule [31] at least 10 of the respondents can be considered experts. Ranking of the seven scenarios by diagnostic value consistently showed that the scenario selected as the most important was the detection of MAML2 rearrangements to confirm the suspected diagnosis of MEC (Table 3). Specifically, this setting received a relative importance index of 0.8, a subjective rank order of 25, and 20 out of 30 possible top priority ratings (Tables S8 and S9). 

Confirmatory MAML2 detection in MEC was also identified as the most important scenario when restricting the rating to those participants with the highest intra-rater reliability (Table S10). The second most important scenario was also confirmatory (i.e., absence of MAML2 to confirm ASC). The other scenarios, including re-classification via MAML2 results, were considered less important (Table 3). Using the Delphi approach, the group agreed upon diagnostic value ranking and descriptions for the various settings. Discussion points and anonymous comments fell into three categories: survey-related comments, opinions about the selected scenarios, and statements about the diagnostic value of MAML2 rearrangements (Table S11).

## 3. Discussion

Herein, we report the diagnostic utility of MAML2 rearrangements in MEC and ASC. Our test order analysis showed that MAML2 testing was applied predominantly for confirmatory rather than diagnostic reasons (ratio 4:1). Modeling diagnostic probabilities and comparison of test performance metrics quantitatively specified the added diagnostic value of MAML2 testing. Using the Delphi method, our 15-expert survey showed that confirmatory use cases consistently ranked the highest. Thus, the added diagnostic value of MAML2 testing was best understood as a composite of a suboptimal (70%) screening test and a highly specific exclusion tool for MEC and ASC. By delineating the test performance of an imperfect biomarker, as one component in a multi-modality diagnostic workup process, we specified an approach to delineate the added value of a rare biomarker when moving from emerging tool to clinical integration.

The study was triggered by a collaboration under the framework of the International Collaboration for Cancer Classification and Research (IC3R) [32]. Briefly, IC3R is a consortium to address challenges in translating research findings into the WHO Classification of Tumours and, therefore, clinical management [33,34,35]. To identify diagnostic biomarkers that can help to distinguish MEC and ASC in histologically difficult cases, a workgroup used systematic review methods to summarize publications on molecular alterations [24]. The systematic review detected several evidence gaps and methodological quality issues, one of which being the lack of context-specific diagnostic performance metrics for MAML2 rearrangements. Research studies primarily focus on well-defined patient subsets; however, as a diagnostic aid, MAML2 testing is applied prospectively during the workup of difficult rather than well-defined cases. In our practice, we offer genotyping as part of routine clinical care when medically necessary [36,37,38,39]. Due to value-based paradigms and financial constraints, we do not offer genotyping in every case (e.g., completely resected MEC). These constraints are well-known to our surgical pathologists, who consequently have limited access to genotyping and apply testing only in cases where it is medically necessary (i.e., diagnostically challenging and/or end-stage cancer cases). In other words, during the IC3R discussion on the lack of published real-world biomarker data on MAML2 testing in challenging cases, we noticed that the utilization pattern of genotyping in our practice forms an ideal setting to assess its clinical utility. 

Our utilization analysis quantified how the integration of molecular findings results in deviations from molecular working diagnoses. The relevance of distinguishing MEC from ASC is depicted in Figure 1. The outcome comparison, shown in almost 20,000 patients from the SEER database, clearly shows the more aggressive behavior of ASC when compared to MEC. Of note, this comparison is often quoted but rarely visualized [40]. However, we caution that comparing population-based data with data from a convenience sample presents important limitations. Histologic similarities, limited samples, or difficult cases, coupled with drastically different outcomes, explain the significant interest in diagnostic biomarkers [3,4,6,41,42,43,44,45,46,47,48,49,50,51]. Notably, MAML2 rearrangements are, however, not restricted to salivary gland MEC. Previous studies described MAML2 rearrangements in MEC of various sites [22,52,53,54], as well as in the metaplastic variant of cystadenolymphoma (Warthin tumor) [55,56,57], although the MAML2 rearranged Warthin tumors may have been misclassified as Warthin-like MEC [57,58,59,60]. These data raise the question of how to capture the value of MAML2 testing [25]. Our utilization analysis showed that when a case with the working diagnosis of MEC shows an absence of MAML2 rearrangement, the test result is (at first glance) considered non-contributory. The absence raises interesting biological questions (e.g., regarding the missing molecular driver in higher grade MEC). However, these considerations do not help from a diagnostic perspective. Importantly, while negativity for MAML2 rearrangements cannot *rule-in* MEC, the absence of a MAML2 rearrangement can be regarded as confirmatory for ASC. We calculated the test performance characteristics when considering the “absence of MAML2” as a diagnostic indicator for ASC (100% sensitive, 62.2% specific; Table S4). Simply put, before dismissing the test result (or value of MAML2 testing overall), it was important to examine the numerical shift in test performance across the various scenarios (Table 2). Specifically, the successively lower sensitivities for MAML2 testing in MEC were not accompanied by alterations in specificity for the absence of MAML2 rearrangements in ASC (Table 1). In other words, the MAML2 rearrangements status was not sensitive for MEC, yet we are highly confident that MAML2 rearrangements were absent in ASC (*n* = 0/155; 95% confidence interval 0–0.024%). The manifestation of transition probabilities is shown in the Markov model (Figure 3b) and surgical pathologists can review pre-test probabilities along specific differential diagnostic pathways when ordering molecular testing. Thus, our utilization data showed that the value of MAML2 testing was a composite of an imperfectly sensitive screening tool for MEC (70%, Table S5) and a highly specific exclusion tool for ASC (100%, Table S5). We proposed the combined average J-index as a numerical capture for value assessment in imperfect, composite biomarkers and the combined average J-index for MAML2 testing was 85% value-added.

When pathologists contribute diagnostic information to deliver the best therapy and care [61], most surgical pathologists strive to provide maximally informative, and whenever possible, definitive diagnoses [62,63,64]. Instead of issuing preliminary or incomplete reports, pathologists use a variety of reporting instruments [65,66,67,68,69,70,71] to maximize the efficiency of information exchange. However, for clinicians, there is a largely underappreciated complexity of decisions that goes into the workup of a case toward a final diagnosis [72]. For example, additional macroscopic sampling, additional levels, special stains, immunohistochemistry, and molecular testing are all incorporated into the clinical picture. Each decision can be seen as a component that contributes to a result in an overarching testing and refuting framework, where some results will be essential and others are considered non-contributory [73,74,75]. In addition, certain biomarkers may have multiple functions (e.g., high-risk HPV testing or mismatch repair assessment) that can provide information about the pathogenesis or have diagnostic implications in some settings, but allow for prognostic or even enable therapeutic decision-making in other settings [28,76,77,78]. Aside from testing, there are utilization patterns, turn-around time pressures, daily case loads, local availability of certain tests, or even intellectual traditions that contribute in various proportions to the final diagnosis and thereby to the overarching diagnostic test performance of the pathologist or practice. It is improbable to accurately describe the quantitative influence of each factor on the overall performance of this multi-dimensional “chained” event cascade, which emphasizes the importance of real-world data (https://www.fda.gov/science-research/science-and-research-special-topics/real-world-evidence, accessed on 2 January 2022). Practically, it seems obvious that the context of use (e.g., application of a biomarker in a challenging case) has implications for the diagnostic test performance. However, approximating this influence from real-world data poses unique challenges. We have carefully tracked these trajectories from a very specific vantage point (MEC vs. ASC and MAML2 test results) and, to our knowledge, the context-related specification of the diagnostic value-added for MAML2 testing has not been previously clarified. We consider the presented approach, i.e., to dissect the context-specific value of one diagnostic biomarker as a component in a multi-modality diagnostic workup, to be a novel approach to harness the power of real-world data to specify the most meaningful use and integration of a novel biomarker. 

The expert ranking obtained via a Delphi survey confirmed the importance of MAML2 testing as a confirmatory biomarker. One challenge of assessing the specific value of MAML2 testing in distinct diagnostic settings is that we had to be very specific. We, therefore, designed specific diagnostic scenarios (Supplementary Materials) and noticed that these appeared, at first glance, artificial. Several comments by the experts confirmed this notion (Table S10). Two experts suggested that a future study could entail an image-based component to further model transition probabilities; however, this approach would likely entail interpretative variability (i.e., capture combined histology and molecular information) rather than the specific added value of MAML2 testing. It is therefore important to point out that the experts ranked scenarios while being blinded to our utilization data, e.g., the 4:1 ratio of confirmatory to diagnostic utilization. Despite the artificial nature of scenarios and being blinded, it is noteworthy that the two top-rated scenarios in the expert ranking made up 56.3% of our cases in test order practice. In other words, two independent lines of evidence—utilization data and expert ranking—established that the value of MAML2 testing was mostly that of a confirmatory biomarker.

The limitations of our study fall into several categories. The study of real-world data is always biased and applying an adequate study design to reduce the risk of bias is not always feasible. Our study sample and test order analysis were derived from a single institution in a tertiary care setting, and it remains questionable whether our findings apply to other settings. Our order practice is prone to a high risk of selection bias because we do not test every case and testing is ordered at the discretion of the provider or surgical pathologist (resulting in cost savings of ~USD 85k). We assume that higher detection rates can be accomplished when testing all cases; however, medical necessity remains questionable. Molecular testing takes time and can delay the finalization of the surgical pathology diagnosis. Therefore, surgical pathologists are disincentivized to order molecular testing and this may skew the observed confirmatory to diagnostic ratio upward. The test order analysis of 55 cases was limited, especially when compared to the thousands of tumors tested. However, when considering other studies of MEC and ASC, the number of 55 cases is relatively high [24]. Nonetheless, there are statistical implications when using small numbers for modeling. Specifically, the size of our data set (when compared to the relative state space that would approximate the ground truth) implied that our empirical estimates were not expected to converge to the true values of the underlying state probabilities. Therefore, the depicted probabilities were specific to our test practice; however, the approach is generalizable. Similarly, the diagnostic test performance metrics when examining challenging cases are relying on much smaller numbers than portrayed in prior studies. Thereby, our data also illustrate the discrepancy between studies designed and conducted in pre-specified research settings with well-defined cohorts. All these limitations apply; however, we considered these contextual and operational aspects an integral component of clinical care and the value proposition of a new biomarker must withstand these constraints.

Diagnostic performance characterization using real-world evidence is integral to the practice of pathology. Molecular testing, machine learning, and artificial intelligence tools are entering clinical practice and taking on tasks while closely supervised by pathologists [79,80]. This means a shift from mental integration of numerous test results, to specifying diagnostic performance metrics of individual, chained tests. Thus, our findings represent an approach to delineate the context-specific diagnostic value of one specific component (MAML2 testing) in a multi-modal process by using real-world utilization data and expert opinion. The biomarker frequency and diagnostic performance in a specific context determine the added diagnostic value and should drive utilization toward meaningful clinical integration.

## 4. Materials and Methods

### 4.1. Study Design

The project was designed as a retrospective analysis of existing data supplemented by an expert survey. Briefly, we applied a mixed-method approach using population-based data, retrospective institutional data from clinical practice, and survey data from an expert group. The project was conducted in a clinical molecular diagnostics laboratory. All patients consented to genetic testing as part of their routine clinical care and the results were reported in the medical record. We obtained institutional review board approval and the research was performed in accordance with the Declaration of Helsinki. 

### 4.2. Clinical Genotyping

In our practice, we developed the anchored multiplex PCR (AMP) technology for the identification of gene fusions [36]. Briefly, after extraction of total nucleic acids from formalin-fixed paraffin-embedded (FFPE) tissue samples, the samples were reverse transcribed. Two hemi-nested PCR reactions created a fully functional sequencing library using custom designed FusionPlex Solid Tumor primers (ArcherDx Inc., Boulder, CO, USA). NextSeq 2 × 150 base paired-end sequencing results (Illumina, San Diego, CA, USA) were aligned to the hg19 human genome reference using bwa-mem [36]. A laboratory-developed algorithm was used for fusion transcript detection and annotation. The fusion assay was clinically validated for solid tumor samples showing 5% or higher tumor purity. 

### 4.3. Data Sources and Queries 

**Figure 4:**
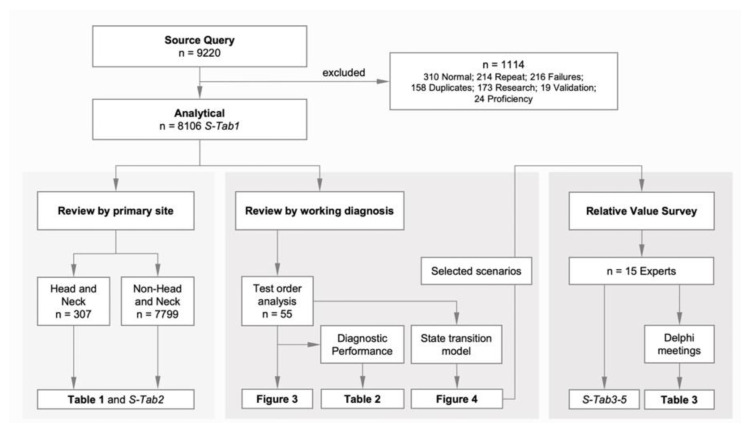
Summary of data acquisition. After data clean-up, the analytical cohort was divided into 2 cohorts (review by primary site and review by working diagnosis).

As a population-based reference and to directly compare overall survival differences in MEC vs. ASC, we queried the National Cancer Institute’s Surveillance, Epidemiology, and End Results (SEER) database for MEC (ICD-O-8430) and ASC (ICD-O-8560) and pulled case listings from 2000–2017. For institutional data analysis, we ran a source query (all cases 29 April 2016–18 June 2020) and excluded non-relevant results to derive the main analytical cohorts (Figure 4). 

### 4.4. Test Order Analysis

When molecular testing is ordered, a working diagnosis is submitted (molecular working diagnosis). For test order analysis, we selected all cases where the molecular working diagnosis contained MEC or ASC and extracted anatomic site and subsite, organ system and location, date of surgery, date of submission for molecular testing, molecular working diagnosis, MAML2 fusion status, fusion partner gene, involved exons, and the date of final (or revised) molecularly informed diagnosis. 

**Figure 5:**
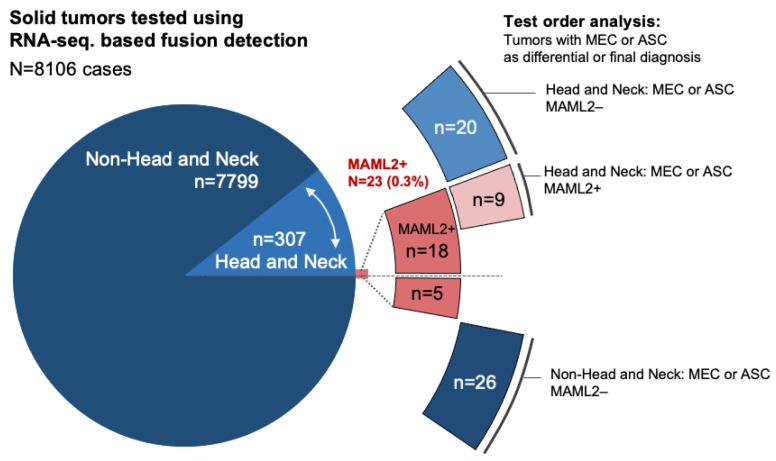
Relationship between analytical, primary site, and test order analysis cohorts. Abbreviations: MEC, mucoepidermoid carcinoma; ASC, adenosquamous carcinoma.

Based on the relationship between the ordering of and the resulting molecular and surgical pathology reports (Figure 2a,b), we distinguished cases where the surgical pathology report was finalized before molecular test results were available (i.e., confirmatory; Figure 2c) from those where the surgical pathologist waited for the molecular results before finalizing the surgical pathology report (i.e., diagnostic; Figure 2d). Cases where molecular MAML2 findings changed between the working diagnosis and the final diagnosis were additionally labeled “molecularly-changed” (Figure 2e,f). The relationship between the different cohorts is shown in Figure 5.

### 4.5. Markov Model

We considered the diagnoses before and after MAML2 results to represent states in a memoryless (Markov) stochastic process where state transitions are determined exclusively by the relationship between pre- and post-molecular diagnoses. To summarize, we computed the empirical estimates of state transition probabilities by conditioning and normalizing the data. We modeled the diagnostic decision-making course starting from 3 working diagnostic states (MEC, ASC, and other (i.e., non-MEC/ASC)) to 4 final diagnostic states (MAML2+ MEC, MAML2− MEC, ASC, and other).

### 4.6. Expert Opinion

As a separate analytical approach, we applied a modified Delphi method consisting of an online survey with three online meetings (January–March 2021). Generally, the Delphi process enables gathering data when there is little or no definitive evidence and where opinion is important. We defined scenarios as the triplet of working diagnosis, MAML2 result, and final diagnosis. We administered a survey incorporating these scenarios and participants were informed about the aim to derive and rank the specific diagnostic value of MAML2 results in each scenario. 

The survey used Likert rating scales and rank order listing to delineate subjective importance (Qualtrics, Provo, UT, USA). Of note, participants were blinded to utilization data and were not informed that rank order and Likert rating used the same scenarios. The meetings were held online and allowed the panelists to provide further clarification and present arguments to justify their points of view. The responses were aggregated and discussed with the group using the multi-step Delphi method to arrive at a group opinion. 

### 4.7. Statistical, Economic Impact, and Survey Analysis

Fusion frequency was defined as the fraction of MAML2 rearranged cases over total informative samples. For visualization, we used a parallel coordinates plot for categorical variables (alluvial diagram, https://observablehq.com/d/2fbc4e269927c907, accessed on 10 February 2021). Statistical performance measures and confidence intervals were determined using the MedCalc toolkit (https://www.medcalc.org/calc/diagnostic_test.php, last accessed 15 March 2022). For the economic impact analysis, we used the clinical laboratory fee schedule amount of USD 597.91 for CPT code 81445. For survey results, we calculated the relative importance index (RII) as the sum of (V)/(A × N), where V is the value-added given on the 0–100 Likert scale to each setting by the respondents, A is the highest value added (i.e., 100 in this case), and N is the total number of respondents. To evaluate the expert ranking of survey settings, we calculated the subjective rank order (SRO) as the sum of each rank by each respondent (range: N to the number of scenarios × N), where the lowest number represents the most important setting. Data were analyzed using Prism 9 (GraphPad Software Inc., San Diego, CA, USA) and Microsoft Excel for Mac V16.48 (Microsoft Corp., Redmond, WA, USA). 

## Figures and Tables

**Table 1:** MAML2 Fusion Frequency in Clinical Practice.

Cases	Total	MAML2+/Total	%	Fusion
All	8106	23/8106	0.28%	
Head and neck	307	18/307	5.86%	
Head and neck/all MAML2+	-	18/23	78.3%	MAML2 (exon2)–CRTC1 (exon1), *n* = 17
				MAML2 (exon2)–CRTC3 (exon1), *n* = 1
Other than head and neck	7799	5/7799	0.06%	
Brain	742	1/742	0.13%	MAML2 (exon2)–YAP1 (exon5), *n* = 1
Breast	414	2/414	0.48%	MAML2 (exon2)–CRTC1 (exon1), *n* = 2
Thymus	16	1/16	6.25%	MAML2 (exon2)–KMT2A (exon10), *n* = 1
Lung	2364	1/2364	0.04%	MAML2 (exon3)–SAMSN1 (exon2), *n* = 1

Abbreviations: MAML2+, MAML2 rearrangement detected. For details, see Tables S1 and S2.

**Table 2:** Four sets of test performance metrics for MAML2 rearrangement as a biomarker for the diagnosis of MEC demonstrated differing patterns by setting.

Setting	Sensitivity (%)	Specificity (%)	PPV (%)	NPV (%)	Youden’s J-Index
Literature	62	100	100	18	0.62
Salivary gland	60	100	100	73	0.60
Test order analysis					
Confirmatory testing *	70	100	100	67	0.70
Confirmatory testing	62	100	100	81	0.62
Diagnostic testing	33	100	100	67	0.33

Abbreviations: MEC, mucoepidermoid carcinoma; NPV, negative predictive value; PPV, positive predictive value; for details see Supplement. * Subtracting *n* = 18 cases with >30-day delay from final sign-out to molecular test order (i.e., therapeutic intent).

**Table 3:** Survey results.

Scenario Working Dx > MDx > Final Dx	RII	RII Rank	SRO	SRO Rank	Description	RWE%	Markov
MEC MAML2+ MEC	0.80	1	25	1	Molecular confirmation	12.7	0.3
ASC MAML2− ASC	0.65	3	40	2	Molecular confirmation *	43.6	0.96
ASC MAML2+ MEC	0.68	2	47	3	Re-classified via molecular	0	0
Other MAML2+ MEC	0.59	4	51	4	Re-classified via molecular	3.5	0.29
MEC MAML2− MEC	0.39	7	59	5	“Molecularly unchanged”	10.9	0.26
Other MAML2− Other	0.51	5	68	6	Molecular confirmation *	7.2	0.57
Other MAML2+ Other	0.43	6	74	7	Molecularly unchanged	0	0

For details see Supplementary Tables S6–S11. Abbreviations: ASC, adenosquamous carcinoma; Dx, diagnosis; Markov, state transition probabilities (Figure 4); MDx, molecular diagnostic test result (here, MAML2 rearrangement status); MEC, mucoepidermoid carcinoma; RII, relative importance index; RWE, real-world evidence; SRO, subjective rank order; other, diagnoses other than ASC or MEC. * Supportive via exclusion of MAML2 rearrangement.

## Data Availability

All data is provided in the Supplementary Materials.

## Supplementary Materials

The following supporting information can be downloaded at: https://www.mdpi.com/article/10.3390/ijms23084322/s1.
